# An empathic approach to recovery

**DOI:** 10.1192/j.eurpsy.2026.10298

**Published:** 2026-07-31

**Authors:** R. Cornish

**Affiliations:** 1 Broadmoor Hospital, West London NHS Trust, Crowthorne; 2 Department of Psychiatry, Oxford University, Oxford, United Kingdom

## Abstract

**Abstract:**

Psychiatric hospitals, and forensic mental health settings face unique challenges in delivering empathic care. Society has wrestled with how to deal with offences perpetrated by the mentally unwell for centuries, and forensic psychiatry has ambiguous guiding principles. At times, the practitioner’s duty is to justice rather than the patient, and detached concern is required to balance competing ethical demands. Challenges are posed by working with patients for whom a lack of empathy can be an integral part of their mental disorder. Forensic mental health settings represent the last long stay environments in England. Lack of scrutiny, high levels of restrictive practice, and frequent violence and aggression, can lead to burnout and failures in compassionate care. Empathic engagement in central to the therapeutic endeavour in forensic settings, to achieve understanding and to enable positive change. This should be supported by reflective spaces, restorative practice, effective outcome measures, system-based approaches to learning and value-driven leadership.

**Disclosure of Interest:**

None Declared

